# Melatonin prevents doxorubicin-induced cardiotoxicity through suppression of AMPKα2-dependent mitochondrial damage

**DOI:** 10.1038/s12276-020-00541-3

**Published:** 2020-12-18

**Authors:** Goowon Yang, Minhyeok Song, Dang Hieu Hoang, Quynh Hoa Tran, Wonchae Choe, Insug Kang, Sung Soo Kim, Joohun Ha

**Affiliations:** Department of Biochemistry and Molecular Medicine, Graduate School, Biomedical Science Institute, Kyung Hee University, Seoul, Korea

**Keywords:** Biochemistry, Diseases

## Abstract

The clinical application of doxorubicin, one of the most effective anticancer drugs, has been limited due to its adverse effects, including cardiotoxicity. One of the hallmarks of doxorubicin-induced cytotoxicity is mitochondrial dysfunction. Despite intensive research over recent decades, there are no effective approaches for alleviating doxorubicin-induced cytotoxicity. Melatonin, a natural hormone that is primarily secreted by the pineal gland, is emerging as a promising adjuvant that protects against doxorubicin-induced cytotoxicity owing to its pharmaceutical effect of preserving mitochondrial integrity. However, the underlying mechanisms are far from completely understood. Here, we provide novel evidence that treatment of H9c2 cardiomyoblasts with doxorubicin strongly induced AMP-activated protein kinase α2 (AMPKα2), which translocated to mitochondria and interfered with their function and integrity, ultimately leading to cellular apoptosis. These phenomena were significantly blocked by melatonin treatment. The levels of AMPKα2 in murine hearts were tightly associated with cardiotoxicity in the context of doxorubicin and melatonin treatment. Therefore, our study suggests that the maintenance of mitochondrial integrity is a key factor in reducing doxorubicin-induced cytotoxicity and indicates that AMPKα2 may serve as a novel target in the design of cytoprotective combination therapies that include doxorubicin.

## Cancer treatment: A chemo combo that protects heart health

The hormone melatonin reduces heart damage caused by a commonly used chemotherapeutic agent, opening the way towards safer cancer treatment. Doxorubicin is a potent killer of tumor cells, but also has toxic effects on cardiac muscle cells, where it severely damages the mitochondria. Melatonin is best known as a regulator of circadian rhythms, but Joohun Ha and colleagues at Kyung Hee University in Seoul, South Korea, have determined that it can also counteract doxorubicin toxicity. Working with cultured heart cells, the researchers showed that doxorubicin stimulates production of a signaling protein called AMPKα2. This protein subsequently enters the mitochondria and disrupts their structural integrity, leading to cell death. However, adding melatonin to treatment with doxorubicin prevents induction of AMPKα2, thereby increasing heart cell survival.

## Introduction

Doxorubicin is one of the most potent chemotherapeutic agents and is widely used for the treatment of various cancers and hematological malignancies. However, the more widespread use of this agent is limited owing to its severe adverse effects, including cardiotoxicity, neurological disturbances, and bone marrow aplasia^1,2^. Decades of studies on doxorubicin have implicated multifactorial processes in the development of doxorubicin-induced cytotoxicity, but recent studies suggest that mitochondrial dysfunction is a distinctive hallmark of doxorubicin-induced cytotoxicity^3,4^.

To alleviate doxorubicin-induced toxicity, researchers have tested a number of strategies, including the administration of antioxidants and/or antiapoptotic agents, in both in vitro and in vivo models of doxorubicin-induced cytotoxicity, but most of these trials have failed to translate into clinical benefits^5–7^. As a result, there are no effective approaches for alleviating doxorubicin-induced cytotoxicity despite intensive research over recent decades. Melatonin is a natural hormone that is primarily secreted by the pineal gland and functions as a major regulator of circadian rhythms in humans^8^. Melatonin also plays a variety of biological roles as a modulator of mood, sexual behavior and sleep; low levels or a deficiency of melatonin are also associated with Parkinson’s disease, Alzheimer’s disease, epilepsy, ischemic injury, diabetes, and even cancer^8,9^. Melatonin has emerged as a promising adjuvant that protects against doxorubicin-induced cytotoxicity, as highlighted by various studies and clinical trials that have demonstrated its cardioprotective effects against several chemotherapeutic agents^10–12^. These beneficial effects of melatonin are largely associated with its ability to prevent mitochondrial dysfunction and its strong antioxidant properties^13–15^. Moreover, melatonin exhibits low toxicity and easily enters cells owing to its good solubility in both aqueous and organic phases and its highly lipophilic properties^13^. Thus, melatonin shows great promise for use in this therapeutic context.

In the present study, we examined the mechanisms by which melatonin attenuates doxorubicin-induced cytotoxicity, revealing a novel role of AMPKα2 in the mitochondria of H9c2 cardiomyoblasts and mouse hearts. AMPK is composed of a catalytic subunit (α) and two regulatory subunits (β and γ), each with multiple isoforms, and AMPK plays a central role in the regulation of energy homeostasis^16–20^. Regarding the catalytic subunit, α1 is ubiquitously expressed in all tissues, but α2 is highly expressed in metabolically active tissues, such as the heart, skeletal muscle, and liver; α2 has also been implicated in the regulation of carbohydrate and lipid metabolism^21–23^. In a previous study, we described a novel role for AMPKα2 in the context of the treatment of mouse embryonic fibroblasts (MEFs) with doxorubicin, showing that AMPKα2 expression is robustly induced by doxorubicin at the transcriptional level via the transcription factor E2F1 and subsequently contributes to apoptosis^24^. However, the features of AMPKα2 that are responsible for these proapoptotic properties remain unknown. Here, we provide novel evidence that AMPKα2 exerts a proapoptotic effect in the context of doxorubicin treatment by interfering with mitochondrial integrity in H9c2 cardiomyoblasts and mouse hearts. Notably, we found that these effects were effectively blocked by melatonin.

## Materials and methods

### Reagents and antibodies

Dulbecco’s modified Eagle’s medium (DMEM), F-12 (1:1), and bovine serum (BS) were purchased from Gibco (Grand Island, NY). Fetal bovine serum (FBS) was purchased from Corning (Corning, NY). Melatonin, doxorubicin, H2DCF-DA, intracellular ATP assay kits, N-acetyl cysteine, and 4-P-PDOT (4-phenyl-2-propionamidotetralin) were purchased from Sigma-Aldrich (St. Louis, MO). MTT thiazolyl blue and D-luciferin were purchased from Duchefa Biochemie (Haarlem, the Netherlands). 4’,6-Diamidino-2-phenylindole (DAPI), enhanced chemiluminescence reagent, antibodies against GAPDH, E2F1, c-Myc, and voltage-dependent anion channel (VDAC), MitoTracker red, and control IgG antibodies were purchased from Santa Cruz Biotechnology (Santa Cruz, CA). Anti-AMPKα2 and anti-AMPKα1 antibodies were purchased from R&D Systems (Minneapolis, MN). MitoTracker green and anti-phospho-H2A.X (S139), anti-phospho-AMPKα (Thr172), anti-phospho-acetyl-CoA carboxylase (ACC) (S79), anti-ACC, anti-phospho-mitochondria fission factor (MFF) (S146), and anti-MFF antibodies were purchased from Cell Signaling Technology (Danvers, MA). MitoSOX Red was purchased from Invitrogen (Carlsbad, CA). The FITC-annexin V apoptosis kit was purchased from DB BioScience (San Diego, CA). The Total OXPHOS Rodent WB Antibody Cocktail was purchased from Abcam (Cambridge, MA).

### Cell culture

H9c2 (rat cardiomyoblast) cells were purchased from American Type Culture Collection (ATCC CRL-1446) in 2012 and maintained in DMEM and F-12 (1:1) supplemented with 10% BS. H9c2 cells fuse to form multinucleated myotubes in response to low serum, and a recent characterization was performed in 2017. Microscopic analysis revealed that these cells formed multinucleated myotubes when cultured in media containing 1% horse serum, and RT-PCR analysis showed an increase in the expression of acetyl-CoA carboxylase β, which is a specific marker of myotube formation^25^. AMPK wild-type (WT) and AMPK knockout MEFs were generated by Benoit Viollet (INSERN, France) in 2006^26^ and generously provided to us in 2008. *Ampkα* WT, *Ampkα2*^−/−^, *Ampkα1*^−*/*−^, and *Ampkα* double knockout (*α1*^*−/−*^*α2*^−*/*−^) MEFs were maintained in DMEM supplemented with 10% FBS. We characterized these cells according to the presence of AMPK isoforms and simian virus 40 large T antigen using Western blot and RT-PCR in 2018. All the cells were supplemented with 1% penicillin/streptomycin and cultured at 37 °C in a humidified environment with 95% air and 5% CO_2_.

### Stable cell transfection

When the density of the H9c2 myocytes reached 50–60%, the cells were transfected with the expression vector pcDNA 3.1 encoding c-Myc-tagged wild-type (WT) or dominant negative (DN, K45A) forms of AMPKα2 using Lipofectamine 3000 (Invitrogen, Carlsbad, CA, USA). The cells were selected for 7 days in the presence of G418 (1 mg/mL), and the selected cells were used for the subsequent experiments.

### DNA plasmids

The promoter region of the human AMPKα2 gene (−2.7 kb) was amplified by PCR from HEK293 genomic DNA and cloned into the pGL3-basic reporter vector. The [E2F]x4-Luc reporter construct was generously provided by Dr. Young-Chae Chang (The Catholic University, School of Medicine, Daegu, Korea). AMPKα2 wild-type (WT) and AMPKα2 dominant-negative (DN, D157A) forms were generated by PCR and cloned into the pCMV vector. The dsRed-mito plasmid was generously provided by Dr. Youngmi Kim Pak (Kyung hee University, School of Medicine, Seoul, Korea). Mito-ABKAR was a gift from Takanari Inoue and Jin Zhang (Addgene plasmid #61509). Tom20-mChF-AIP and mChF-AIP were gifts from Takanari Inoue (Addgene plasmids #61512 and #61527).

### Cell viability assay

Cell viability was measured in 96-well plates using a quantitative colorimetric assay with 3-(4,5-dimethylthiazol-2-yl)-2,5-diphenyltetrazolium bromide (MTT), which is an indicator of mitochondrial activity in live cells. Briefly, 30 μL MTT (final concentration, 0.5 mg/mL) was added to the medium at the indicated times after treatment and then incubated at 37 °C for 4 h. The MTT solution was removed, and 100 μL DMSO was added to each well. The absorbance of each well was measured at 540 nm using a microplate reader (BioTek, Winooski, VT).

### Western blot analysis and immunoprecipitation

Cells were lysed in lysis buffer (50 mM Tris–HCl, pH 7.4, 150 mM NaCl, 1% NP40, 2 mM EDTA, 10 mM NaF, 2 mM Na3VO4). The protein concentrations in the total lysates were determined by the Bradford method (Bio–Rad, Hercules, CA, USA). Twenty micrograms of protein from the total lysates was subjected to SDS-PAGE and transferred to Immobilon-P membranes (Millipore, Bedford, MA). Then, the membranes were incubated with primary antibodies in blocking solution (5% skim milk). The proteins were visualized using the ECL detection system. For the immunoprecipitation experiments, cell lysates were precleared with protein G/A beads and subsequently incubated for 1–2 h with protein G/A beads covalently coupled with anti-AMPKα2 or anti-c-Myc. The immune complexes were washed three times with cell lysis buffer. The eluted samples and whole lysates were resolved by SDS-PAGE, and the proteins were detected by western blot using the indicated antibodies. Quantification of western blots was performed by using ImageJ software.

### Luciferase assay

H9c2 cells were cotransfected with 0.5 μg of the reporter vector. The empty pcDNA vector was used to adjust the total amount of DNA, and 0.5 μg of pCMV-EGFP expression plasmid was used as the internal control. Luciferase activity was determined using a luciferase assay kit (Promega) according to the manufacturer’s instructions. The relative luciferase activity was normalized against the GFP fluorescence intensity.

### Fluorescence-activated cell sorting (FACS) analysis

Fluorescence was determined by flow cytometry (Beckman, Pasadena, CA). MitoTracker green, H2DCF-DA, and Annexin V-FITC fluorescence was measured by excitation at 488 nm and emission collection at 525 nm. Propidium iodide (PI), MitoTracker red, and MitoSOX Red fluorescence was measured by using excitation and emission wavelengths of 540 and 580 nm, respectively.

### Confocal microscopy

H9c2 cells were plated on glass coverslips and treated the following day with doxorubicin, with or without melatonin. The cells were fixed by incubating with 4% paraformaldehyde for 20 min and washed with phosphate-buffered saline (PBS). After permeabilization with 0.1% Triton X-100 in PBS for 3 min at room temperature, the cells were incubated with a blocking solution for 30 min at 37 °C. Solutions of anti-AMPKα2 (1:500) primary antibodies were added to the cells and incubated overnight at 4 °C. Thereafter, the cells were washed with PBS and then incubated with 1:500 dilutions of FITC- or Texas red-conjugated secondary antibodies for 2 h at 37 °C. MitoTracker was added before fixation, and DAPI was added after incubation with secondary antibodies. For the Förster resonance energy transfer (FRET) experiments, H9c2 cells were transfected with the mito-ABKAR plasmid and then treated the following day with doxorubicin and/or melatonin. Cyan fluorescent protein (CFP) was excited at 433 nm, and its emitted fluorescence was collected at 480 nm. FRET images were obtained by exciting yellow fluorescent protein (YFP) at 508 nm and collecting its emitted fluorescence at 527 nm. The lengths of mitochondria were calculated from each YFP image using ImageJ software (image.nih.gov/ij/), as previously described^27^, and the FRET fluorescence intensity was analyzed. Images of the cells were acquired using an LSM 510 confocal microscope (Carl Zeiss, Jena, Germany).

### RT-PCR

Total RNA was extracted from cells using TRIzol reagent (Invitrogen). cDNA was synthesized from 2 mg of total RNA using M-MLV reverse transcriptase (Fermentas, Hanover, MD, USA). The specific primers used for RT-PCR were as follows: AMPKα2, 5′-AGC TCG CAG TGG CTT ATC AT-3′ (sense) and 5′-GGG GCT GTC TGC TAT GAG AG-3′ (anti-sense); and GAPDH, 5′-AGA CAG CCG CAT CTT CTT GT-3′ (sense) and 5′-CTT GCC GTG GGT AGA GTC AT-3′ (anti-sense). The PCR products were analyzed by agarose gel electrophoresis and visualized using ethidium bromide. The signals were quantified using ImageJ software (National Institutes of Health (NIH), Bethesda, MD, USA).

### Measurement of mitochondrial DNA content

Total genomic DNA was extracted from H9c2 cells and used as a template for the amplification of mitochondrially encoded cytochrome C oxidase II (MTCO2) and 18S rRNA by real-time quantitative polymerase chain reaction (qPCR). The following specific primer pairs were used: MTCO2, 5’-GCT TAC AAG ACG CCA CAT CA-3’ (sense) and 5′-GAA TTC GTA GGG AGG GAA GG-3′ (anti-sense); 18S rRNA, 5′-CGC GGT TCT ATT TTG TTG GT-3′ (sense) and 5′-AGT CGG CAT CGT TTA TGG TC-3′ (anti-sense). qPCR was performed using a 7500 Real-Time PCR System (Applied Biosystems, Branchburg, NJ, USA) with SYBRGreen PCR Master Mix (Applied Biosystems). A total of 40 thermal cycles were used for the PCR, and the expression of MTCO2 was analyzed using an absolute quantification method and normalized to the levels of 18S rRNA.

### Measurement of intracellular ATP levels

Cells were seeded in 24-well plates and stimulated as indicated. The intracellular ATP content was measured using an ATP Determination Kit (Sigma-Aldrich) in accordance with the manufacturer’s instructions.

### Animal experiments

All the mice were purchased from Japan SLC (Shizuoka, Japan) and housed at 21 ± 2 °C and 50% ± 5% relative humidity with a 12-h light/12-h dark cycle. The mice were randomly divided into 4 groups (*n* = 5/group): sham, doxorubicin, melatonin, and doxorubicin + melatonin. Melatonin (1 mg/kg) and/or doxorubicin (2.5 mg/kg) were intraperitoneally injected into 4-week-old mice (Slic: ICR, male) every 2 days for 2 weeks. After 2 weeks of treatment, heart tissues were isolated, and five-micron-thick sections were subjected to H&E staining, Masson trichrome staining, immunohistochemical analysis (IHC) of AMPKα2, and colorimetric TUNEL assay (Promega Corp., Madison, WI). The thickness of the myofibrillum was measured as previously described^28^. The TUNEL-positive nuclei (dark brown) in the heart tissues were detected using a normal white light microscope (Olympus, Tokyo, Japan). Quantification was performed by counting the number of TUNEL-positive cells in at least five random fields. The animal protocol was approved by the Institutional Animal Care and Use Committee of Kyung Hee University (KHSASP-19-297).

### Data and statistical analysis

The results are expressed as the means ± SEMs. The statistical significance of the data were analyzed by one-way analysis of variance (ANOVA) using the R program suite (version 3.2.4; http://www.r-project.org). By convention, a *p*-value < 0.05 was considered statistically significant. Individual *p*-values are indicated in the figures (*p < 0.05; **p < 0.01). Each experiment was repeated at least twice with three samples each to ensure statistical significance.

## Results

### Melatonin blocks doxorubicin-induced AMPKα2 expression, which has proapoptotic properties in H9c2 cardiomyoblasts

**Fig. 1:**
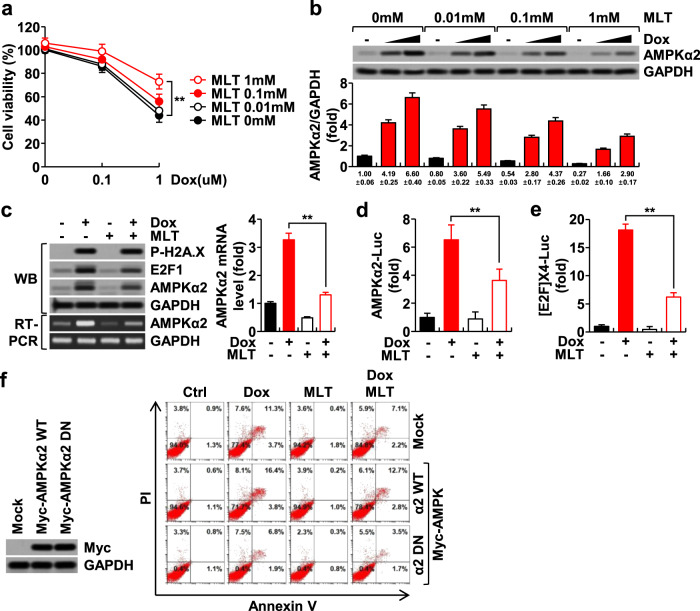
Effects of doxorubicin and melatonin on AMPKα2 and apoptosis in H9c2 cardiomyoblasts. H9c2 cardiomyoblasts were treated with doxorubicin (DOX) and melatonin (MLT) for 24 h. Cell viability assays (**a**), western blotting (**b**), RT-PCR (**c**), and luciferase reporter assays for AMPKα2 promoter activity (**d**) or E2F activity (**e**) were performed. FACS analysis of Annexin V-PI double-positive H9c2 cells overexpressing AMPKα2WT or AMPKα2DN (**f**).

We first examined H9c2 cardiomyoblast cell viability following treatment with doxorubicin and melatonin. The results showed that melatonin alone did not cause toxicity, but it significantly inhibited doxorubicin-induced cytotoxicity (Fig. 1a). In a previous study, we observed that doxorubicin simultaneously induced the expression of AMPKα2, E2F1, and apoptosis markers, including cleaved PARP and caspase-3, within 24 h in H9c2 cells^24^. Melatonin also blocked the induction of AMPKα2 by doxorubicin (Fig. 1b), which occurred at the transcriptional level, as evidenced by increases in AMPKα2 mRNA levels and promoter activity (Fig. 1c, d). Consistent with this finding, doxorubicin also induced the expression of E2F1, an upstream transcription factor for AMPKα2 (Fig. 1c, e). Overexpression of WT AMPKα2 (AMPKα2WT) potentiated doxorubicin-induced apoptosis, whereas a dominant-negative form of AMPKα2 (AMPKα2DN) suppressed this phenomenon (Fig. 1f), indicating that AMPKα2 possesses proapoptotic properties under these conditions, in accordance with a previous report^24^. Collectively, these results indicate that doxorubicin induces AMPKα2, which has proapoptotic properties, and that melatonin suppresses this expression. Throughout the subsequent studies, cells were treated with doxorubicin (1 μM) and melatonin (1 mM) for 24 h.

### AMPKα2 translocates to mitochondria and disrupts their function in response to doxorubicin in H9c2 cells and MEFs, and melatonin blocks these phenomena

**Fig. 2:**
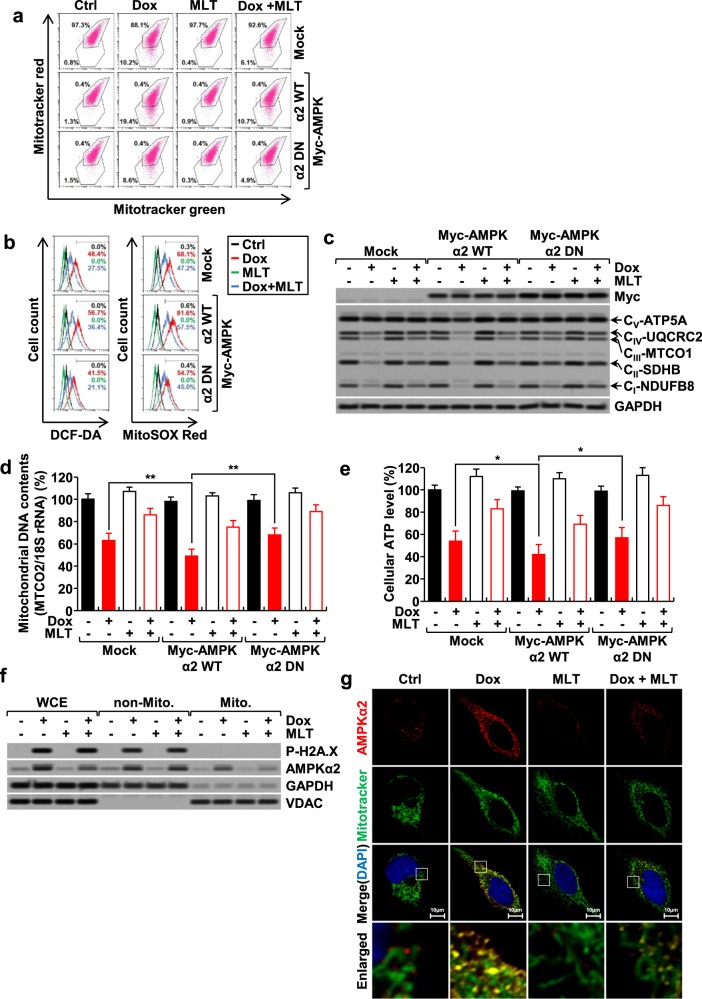
Effects of doxorubicin and melatonin on AMPKα2 and mitochondrial function in H9c2 cardiomyoblasts. H9c2 cells overexpressing AMPKα2WT or AMPKα2DN were treated with doxorubicin (1 μM) and melatonin (1 mM) for 24 h. Under these conditions, mitochondrial membrane potential (**a**), cellular and mitochondrial ROS levels (**b**), mitochondrial OXPHOS complex protein levels (**c**), mitochondrial DNA contents (**d**), and cellular ATP levels (**e**) were measured. The level of AMPKα2 in whole-cell extracts (WCE), nonmitochondrial (non-Mito), and mitochondrial fractions (Mito) (**f**). Fluorescence images of AMPKα2, mitochondria, and nuclei, which were labeled with Texas Red dye, MitoTracker green, and DAPI, respectively (**g**).

We next examined whether AMPKα2 plays a specific role in the regulation of mitochondrial function and integrity. Doxorubicin caused severe mitochondrial damage in H9c2 cells, as indicated by mitochondrial membrane potential (Fig. 2a), cellular and mitochondrial ROS levels (Fig. 2b), mitochondrial OXPHOS complex protein levels (Fig. 2c), mitochondrial DNA content (Fig. 2d), and cellular ATP levels (Fig. 2e). Each of these signs of mitochondrial damage was effectively blocked by melatonin. Under these conditions, the overexpression of AMPKα2WT potentiated the mitochondrial damage induced by doxorubicin, whereas AMPKα2DN reduced this mitochondrial damage. Western blot analysis of mitochondrial fractions (Fig. 2f) and confocal microscopy analysis (Fig. 2g) revealed that a significant portion of AMPKα2 was translocated to mitochondria following doxorubicin treatment and that this translocation was blocked by melatonin.

**Fig. 3:**
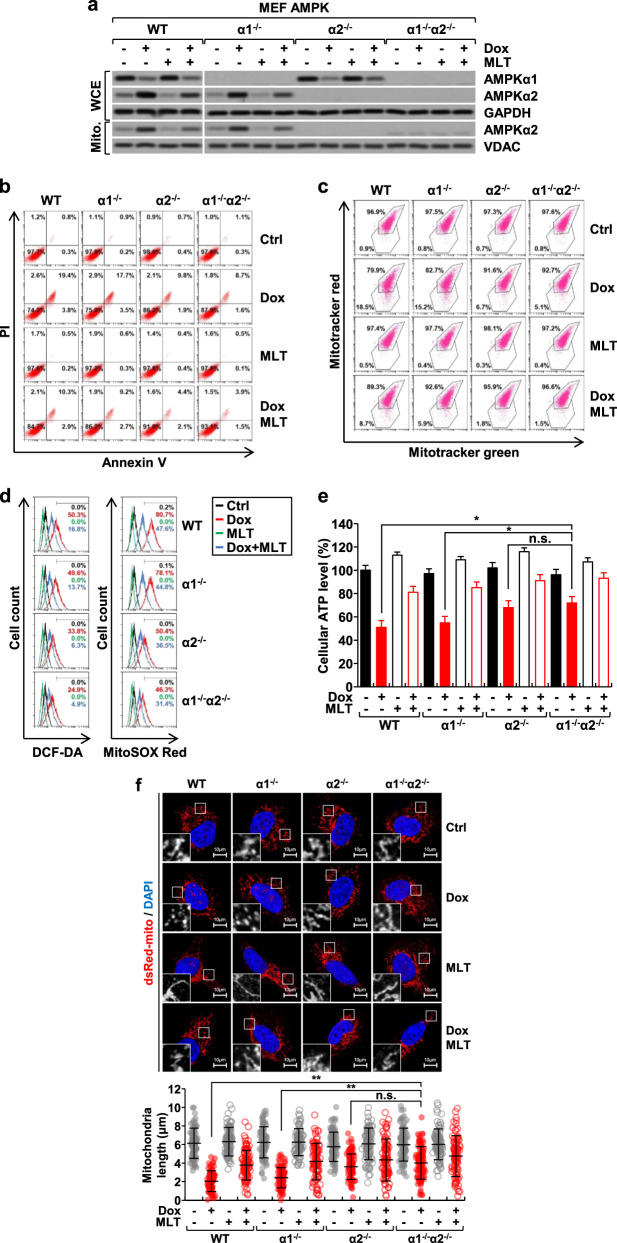
Effects of doxorubicin and melatonin on AMPKα2 and mitochondrial function in AMPK knockout MEFs. WT, *Ampkα1*^*−/−*^, *Ampkα2*^*−/−*^, and *Ampkα1*^*−/−*^*α2*^*−/−*^ double-knockout mouse embryonic fibroblasts (MEFs) were treated with doxorubicin (1 µM) and melatonin (1 mM) for 24 h. The protein levels of AMPKα1 and AMPKα2 in whole-cell extracts (*WCE*) and mitochondrial fractions (*Mito*) (**a**), apoptosis rate (**b**), mitochondrial membrane potential (**c**), cellular and mitochondrial ROS levels (**d**), cellular ATP levels (**e**), and mitochondrial length (f) were measured. The pDsRed-Mito vector was used to label mitochondria (**f**). n.s., not significant.

We further examined the specific role of AMPKα2 in the mitochondria of WT, *Ampkα1*^*−/−*^, *Ampkα2*^*−/−*^, and *Ampkα1*^*−/−*^*α2*^*−/−*^ double-knockout MEFs. The total cellular levels of AMPKα2, as well as the levels of AMPKα2 in the mitochondrial fraction, were increased by doxorubicin in the WT and *Ampkα1*^*−/−*^ MEFs, and melatonin significantly reduced the cellular and mitochondrial levels of AMPKα2 (Fig. 3a). Therefore, comparisons of AMPKα2-positive MEFs (WT and *Ampkα1*^*−/−*^) and AMPKα2-negative MEFs (*Ampkα2*^*−/−*^ and *Ampkα1*^*−/−*^*α2*^*−/−*^) provide an excellent approach for investigating the specific role of AMPKα2 in the context of doxorubicin treatment. In accordance with a previous result (Fig. 1f), the doxorubicin-induced apoptosis and mitochondrial damage were more severe in the AMPKα2-positive MEFs than in the AMPKα2-negative MEFs, as demonstrated by annexin V/PI double staining (Fig. 3b), mitochondrial membrane potential (Fig. 3c), cellular and mitochondrial ROS levels (Fig. 3d), and cellular ATP levels (Fig. 3e). Mitochondria are highly dynamic organelles that maintain their structure and function by undergoing fission and fusion with each other. However, during apoptosis, mitochondria often fragment into smaller units, and the imbalance between fusion and fission is closely related to the degree of mitochondrial dysfunction^29,30^. Treatment of AMPKα2-positive MEFs with doxorubicin caused fragmentation of mitochondrial structures, a phenomenon that was significantly reduced in AMPKα2-negative MEFs (Fig. 3f). Collectively, these results suggest that, in the context of doxorubicin treatment, a significant portion of AMPKα2 is translocated to mitochondria, disrupting mitochondrial function and morphology and ultimately contributing to apoptosis and that melatonin preserves mitochondrial function and integrity by suppressing AMPKα2 expression (Figs. 2 and 3).

### Melatonin prevents doxorubicin-induced mitochondrial fragmentation by suppressing the activity of mitochondrial AMPKα2

**Fig. 4:**
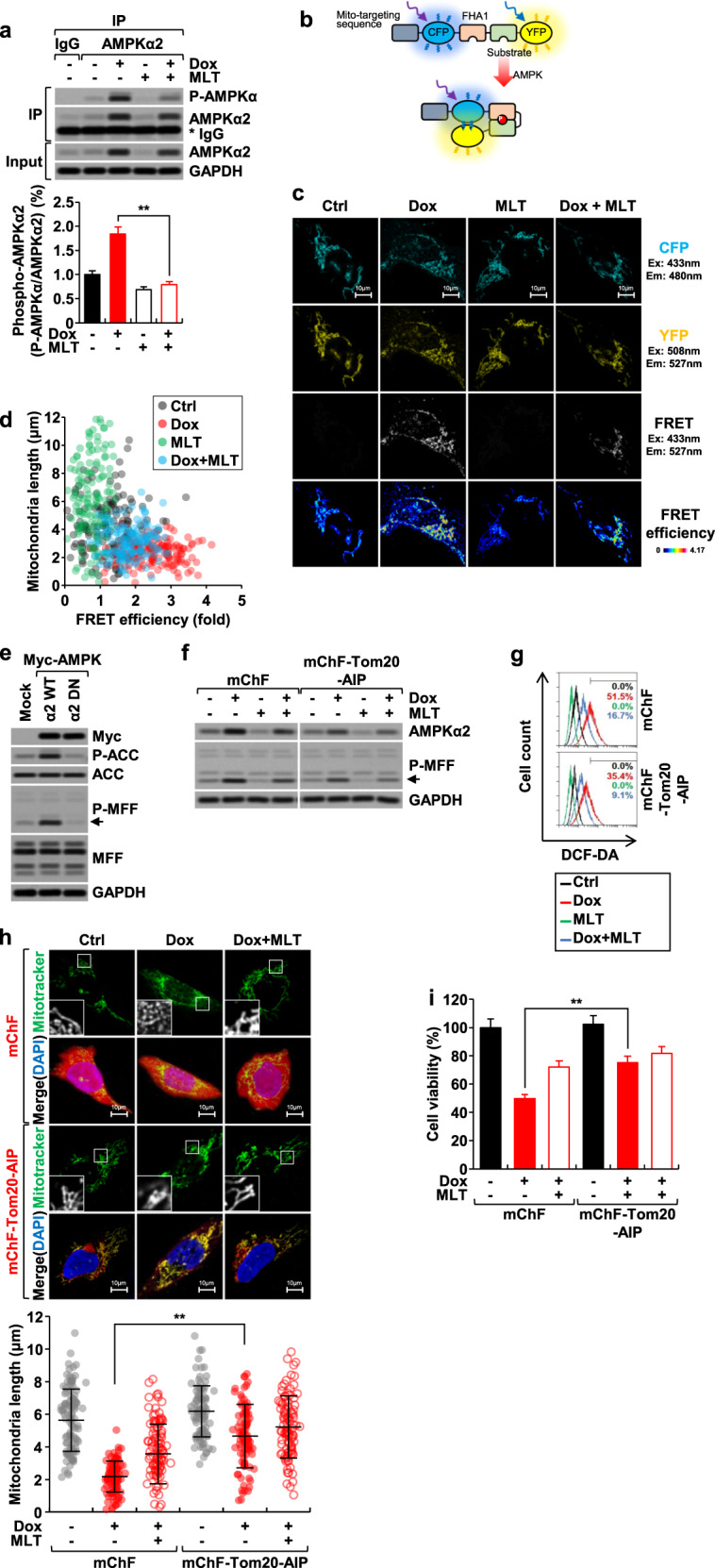
Effects of doxorubicin and melatonin on mitochondria-specific AMPKα2 and mitochondrial function in H9c2 cardiomyoblasts. H9c2 cells were treated with melatonin (1 mM) and/or doxorubicin (1 µM) for 24 h. **a** Endogenous AMPKα2 was immunoprecipitated and analyzed by western blotting. **b** The structure of mito-ABKAR. **c** After transfection with the mito-ABKAR plasmid, representative CFP images, YFP images, and FRET images were obtained by confocal microscopy under the indicated conditions. FRET efficiency was represented as pseudocolor images of FRET/CFP. **d** The correlation between mitochondrial length and FRET signal in the context of melatonin and doxorubicin treatment. **e** MFF phosphorylated at Ser^146^ (P-MFF) in H9c2 cells transfected with AMPKα2WT & DN. P-MFF (**f**), cellular ROS level (**g**), mitochondrial length (**h**), and cell viability (**i**) were measured in H9c2 cells transfected with mChF-Tom20-AIP.

Despite the findings presented above, it remains unclear whether the enzymatic activity of AMPKα2 is actually critical for doxorubicin-induced mitochondrial damage. Analysis of immunoprecipitated AMPKα2 revealed that treatment with doxorubicin significantly increased its activity, and this phenomenon was effectively blocked by melatonin, as determined by the ratio of phosphorylated (active) AMPKα2 to total AMPKα2 (Fig. 4a). We further measured the mitochondria-specific AMPKα2 activity by transfecting the mito-ABKAR plasmid, which is a mitochondria-specific AMPK activity probe. This construct, which is composed of a mitochondria-targeting sequence, the CFP variant Cerulean, the FHA1 phospho-amino acid-binding domain, an AMPK substrate motif, and the YFP variant cpVE172, is a Förster resonance energy transfer (FRET)-based reporter designed to measure the mitochondria-specific activity of AMPK^31^ (Fig. 4b). Doxorubicin treatment induced a strong FRET signal, and this effect was significantly attenuated by melatonin (Fig. 4c). We further observed a strong negative correlation between mito-ABKAR FRET signals and mitochondrial length following treatment with doxorubicin and/or melatonin (Fig. 4d). Relevant to our current study, a recent study demonstrated that AMPK phosphorylated mitochondrial fission factor (MFF), a mitochondrial outer-membrane protein that is required for mitochondrial fission, at Ser^146^ ^32,33^. We observed that overexpression of AMPKα2WT, but not AMPKα2DN, strongly induced the phosphorylation of MFF at Ser^146^ (Fig. 4e). Doxorubicin treatment also strongly induced the phosphorylation of MFF at Ser^146^, whereas melatonin blocked this phosphorylation (Fig. 4f). To further demonstrate the mitochondria-specific role of AMPKα2, we next used the recently developed construct mChF-Tom20-AIP. This construct was designed to express AMPK inhibitor peptide, termed AIP, which allows for the inhibition of AMPK activity in the mitochondria in a substrate-competitive manner^31^. The transfection of mChF-Tom20-AIP into H9c2 cells resulted in a significant reduction in the doxorubicin-induced phosphorylation of MFF at Ser^146^, indicating the specific inhibition of AMPKα2 in the mitochondria (Fig. 4f). Moreover, mChF-Tom20-AIP significantly blocked doxorubicin-induced ROS generation (Fig. 4g), mitochondrial fragmentation (Fig. 4h), and cytotoxicity (Fig. 4i). These results suggest that mitochondria-specific AMPKα2 induces mitochondrial fragmentation in response to doxorubicin and that melatonin attenuates doxorubicin-induced cytotoxicity by suppressing mitochondrial-specific AMPKα2 activity and expression.

### ROS play a key role in AMPKα2 regulation in the context of doxorubicin and melatonin treatment

**Fig. 5:**
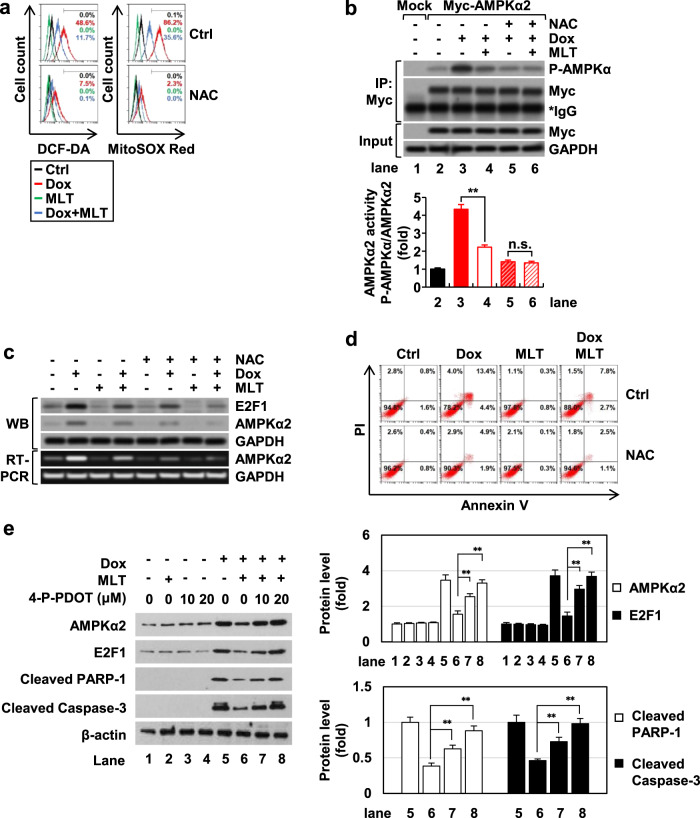
Role of ROS in the induction of AMPKα2 in the context of doxorubicin and melatonin treatment. H9c2 cells were treated with doxorubicin and melatonin in the presence of NAC (5 mM). Cellular and mitochondrial ROS levels (**a**), western blot analysis of immunoprecipitated Myc-AMPKα2 after transfection into H9c2 cells (**b**), western blot and RT-PCR (**c**), apoptosis rate (**d**) were measured or performed. **e** H9c2 cells were treated with doxorubicin and melatonin in the presence of the indicated concentration of 4-P-PDOT, and western blot analyses were performed.

The mechanisms by which doxorubicin modulates AMPKα2 activity or expression are currently unknown. Since doxorubicin induces ROS generation^3^ and AMPK is highly sensitive to intracellular ROS^17^, we examined whether ROS are involved in the regulation of AMPKα2 in the context of doxorubicin and/or melatonin treatment. Pretreatment with an antioxidant, *N*-acetyl cysteine (NAC), significantly blocked cellular and mitochondrial ROS generation (Fig. 5a), AMPKα2 activation (Fig. 5b), AMPKα2 mRNA transcription (Fig. 5c), and apoptosis (Fig. 5d) in the context of doxorubicin treatment. The effects of melatonin on doxorubicin-induced AMPKα2 activity were almost completely diminished in the presence of NAC (Fig. 5b), suggesting that the effects of melatonin, which attenuates doxorubicin-induced AMPKα2 activation, are largely associated with its strong antioxidant properties. NAC treatment decreased doxorubicin-induced E2F1, AMPKα2, and apoptosis as effectively as melatonin, but a combination treatment of NAC and melatonin showed synergistic suppression of these parameters (Fig. 5c, d). Therefore, in addition to its antioxidant properties, melatonin may affect the signaling pathways upstream of AMPKα2 transcription and apoptosis. To test whether melatonin affects these pathways via its receptor, we examined the effects of the highly selective antagonist 4-P-PDOT on MT_2_ (melatonin membrane receptor 2) because H9c2 cells express high levels of MT_2_^34^. In the presence of 4-P-PDOT, the effects of melatonin on the doxorubicin-induced levels of AMPKα2, E2F1, and apoptosis markers were significantly reduced (Fig. 5e). Collectively, our data suggest that melatonin inhibits AMPKα2 enzyme activity and expression due to its ROS-scavenging properties while simultaneously suppressing the expression of AMPKα2 and E2F1 via the MT2-mediated pathway in the context of doxorubicin treatment.

### The expression level and mitochondria-specific activity of AMPKα2 are tightly associated with cardiotoxicity in mice treated with doxorubicin and melatonin

**Fig. 6:**
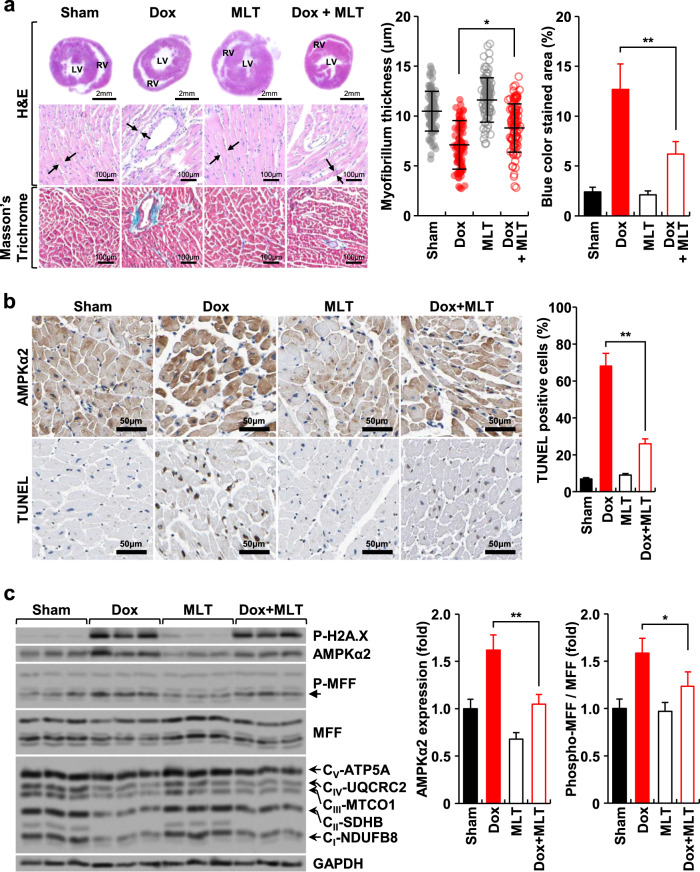
AMPKα2 in the heart tissue of mice treated with doxorubicin and/or melatonin. Mice were intraperitoneally injected with doxorubicin (2.5 mg/kg) and/or melatonin (1 mg/kg) every 2 days for 2 weeks. **a** After performing the experiments, the heart tissues of the mice were extracted, and H&E and Masson’s trichrome staining were performed. The arrows indicate the thickness of the myofibrillum. **b** The expression of AMPKα2 and the number of TUNEL-positive cells in heart tissue were identified by immunohistochemical staining. **c** Western blot analysis of the proteins from the heart tissue of each mouse group. Each image and western blot band were quantified using ImageJ software. LV, left ventricle; RV, right ventricle.

Hematoxylin and eosin (H&E) and Masson trichrome staining of cardiac tissues from doxorubicin-treated mice revealed disorganization of muscle fibers, thinner cardiomyocytes, and increased fibrotic areas, including perivascular areas (Fig. 6a). In the doxorubicin-treated group, the expression level of AMPKα2 and rate of TUNEL positivity in cardiac tissues were significantly higher than those in the control group (Fig. 6b). These changes were effectively blocked by melatonin treatment. Western blot analysis of the proteins in cardiac tissue showed that doxorubicin induced the levels of AMPKα2 and P-MFF but reduced the levels of mitochondrial OXPHOS proteins, and these changes were significantly diminished by melatonin treatment (Fig. 6c). Collectively, the in vivo data support the proposed role of AMPKα2 demonstrated in H9c2 cells in the context of doxorubicin and melatonin treatment.

## Discussion

Doxorubicin effectively kills proliferating cancer cells by intercalating into their DNA and forming covalent adducts. Doxorubicin also accumulates at high concentrations in mitochondria owing to its high affinity for cardiolipin, which is a major phospholipid in the inner membrane of mitochondria; the formation of irreversible interactions between doxorubicin and cardiolipin is known to be an initial cause of mitochondrial dysfunction^35^. This phenomenon is particularly pronounced in cells such as cardiomyocytes, which contain abundant mitochondria. Thus, mitochondrial dysfunction has become a hallmark of doxorubicin-induced cardiotoxicity^3,4^. In addition, doxorubicin activates multiple intracellular signaling pathways that lead to mitochondrial dysfunction and apoptosis^35^. Mitochondria are also known to be a major target of melatonin, which readily crosses all biological membranes because of its amphiphilic nature and thus becomes highly concentrated in mitochondria and nuclei^11^. In addition, melatonin activates multiple signaling pathways that lead to the preservation of mitochondria. It has been reported that melatonin increases the activity of mitochondrial respiratory complexes I and IV, thereby increasing ATP production^36,37^. Melatonin possesses strong ROS-scavenging activity, but it also induces the expression of various antioxidant genes, including glutathione peroxidase, superoxide dismutase, and catalase, while inhibiting the expression of nitric oxide synthase^4^. These beneficial effects of melatonin are expected to effectively mitigate the cytotoxicity of doxorubicin; indeed, the results of various clinical trials suggest that melatonin significantly reduces doxorubicin-induced cardiotoxicity. Thus, the preservation of mitochondrial integrity is a key focus of efforts to develop therapeutic interventions for doxorubicin-induced cytotoxicity^10–12^.

The results of the present study suggest that AMPKα2 is a novel and common target of doxorubicin and melatonin action. In general, AMPKα2 exhibits a distinctive tissue-specific expression pattern, with high expression in skeletal and heart muscle; it also regulates critical metabolic processes, including glucose tolerance, insulin sensitivity, and weight gain^21–23^. Notably, we observed that AMPKα2 exhibits proapoptotic properties in doxorubicin-treated H9c2 cells and MEFs (Figs. 1f, 3b, 4i), in accordance with our previous report^24^. The proapoptotic nature of AMPKα2 is likely attributable to its activity in mitochondria. Our results showed that doxorubicin regulated AMPKα2 at least at three levels. First, doxorubicin significantly increased AMPKα2 expression at the transcriptional level via E2F1 (Fig. 1), which is a well-known transcription factor that mediates doxorubicin-induced cytotoxicity by inducing a number of genes involved in the apoptotic pathway. Notably, in this context, AMPKα2 was recently shown to be a novel target of E2F1^24^. Second, a significant portion of AMPKα2 translocated to mitochondria in response to doxorubicin treatment (Fig. 2f, g). Third, doxorubicin increased the mitochondria-specific enzyme activity of AMPKα2, which contributed to mitochondrial dysfunction (Fig. 4). Although the underlying mechanisms by which doxorubicin causes these changes in AMPK are practically unknown, our data suggest that ROS are a critical factor (Fig. 5). Thus, a vicious cycle between ROS and AMPKα2 is likely to contribute to doxorubicin-induced cytotoxicity. Specifically, ROS cause multiple changes in AMPKα2 in doxorubicin-treated cells, and AMPKα2, in turn, interferes with mitochondrial integrity, resulting in the generation of additional ROS. The effects of melatonin, which attenuates doxorubicin-induced cytotoxicity, are likely associated with its strong antioxidant properties that suppress the vicious cycle between ROS and AMPKα2. Additionally, melatonin may regulate the upstream signaling pathways that lead to AMPKα2 transcription via the MT2-mediated signaling pathway.

The relationship between AMPK and mitochondria has been of great interest to researchers in the energy homeostasis field, but most of these previous studies were performed without distinguishing among the potentially different roles of the various AMPK isoforms. Therefore, some results appear to be quite contradictory. For example, in contrast to the proapoptotic properties of AMPKα2 described in the current study, a recent publication reported that AMPKα, presumably AMPKα1, mediated the protective effects of melatonin against doxorubicin-induced mitochondrial oxidative damage^38^. Another report concluded that AMPKα1 was necessary for the expression of mitochondrial electron transport complex I genes and thereby mitigated metabolic stress and apoptosis in T-ALL cells^39^. More relevant to our current study, mitochondria fission factor (MFF), a mitochondrial outer-membrane protein that is required for mitochondrial fission, was recently identified as a novel substrate for AMPK^32^. We also observed that doxorubicin and AMPKα2 induced the phosphorylation of MFF at Ser^146^, whereas melatonin blocked this phosphorylation (Fig. 4e, f). Notably, in this context, we observed that the expression level of AMPKα2 increased in response to doxorubicin, whereas that of AMPKα1 decreased, and melatonin treatment resulted in the reversal of each of these expression patterns (Fig. 3a). Therefore, AMPKα isoforms may exert opposing effects by differentially regulating the fate of mitochondria under different conditions. Thus, a clearer understanding of the relationship between AMPK and mitochondria requires the consideration of the isoform-specific roles of AMPKα as well as the mitochondria compartment-specific roles of AMPK. In addition, since the enzyme activity of AMPKα2 can be regulated by doxorubicin and melatonin (Fig. 5b), it would be highly interesting to study how the mechanisms upstream of AMPK, including LKB1 (liver kinase B1) and CaMKKβ (calcium/calmodulin-dependent protein kinase kinase β), are regulated under these conditions.

Collectively, our data suggest that the preservation of mitochondrial integrity by melatonin is a critical feature of the ability of melatonin to mitigate doxorubicin-induced cytotoxicity and that AMPKα2 may serve as a novel target in the design of cytoprotective combination therapies that include doxorubicin.
